# Pu-erh Tea Water Extract Mediates Cell Cycle Arrest and Apoptosis in MDA-MB-231 Human Breast Cancer Cells

**DOI:** 10.3389/fphar.2017.00190

**Published:** 2017-04-06

**Authors:** Jing Xie, Haishuang Yu, Shuang Song, Chongye Fang, Xuanjun Wang, Zhongbin Bai, Xiao Ma, Shumei Hao, Hong-Ye Zhao, Jun Sheng

**Affiliations:** ^1^Key Laboratory of Pu-er Tea Science, Ministry of Education, Yunnan Agricultural UniversityKunming, China; ^2^College of Animal Science and Technology, Yunnan Agricultural UniversityKunming, China; ^3^Key Laboratory of Agricultural Biodiversity and Plant Disease Management of China Education Ministry, Yunnan Agricultural UniversityKunming, China; ^4^State Key Laboratory for Conservation and Utilization of Bio-Resources, Yunnan UniversityKunming, China

**Keywords:** Pu-erh tea water extract, apoptosis, cell cycle arrest, JNK, MDA-MB-231 cells

## Abstract

Pu-erh tea is believed to have health benefits, the growth inhibition activity of Pu-erh tea on breast cancer cell has not been investigated. In this study, we examined the activity of Pu-erh tea water extract on apoptosis and cell cycle arrest in the human breast adenocarcinoma cell line MDA-MB-231 and clarified its underlying mechanism of action. We found that Pu-erh tea extract inhibited cell proliferation and induced apoptosis in a dose-dependent manner. We also found that Pu-erh tea extract inhibited tumor cell growth within 24 h via accumulation of cells in S phase. Further experiments showed that at 24 h, Pu-erh tea extract up-regulated the expressions of P-p53 (Ser15), p21 and P-JNK and down-regulated the expressions of PCNA, CyclinD1 and CyclinE at the protein level in MDA-MB-231 cells. In particular, the JNK-specific inhibitor SP600125 restored the induction of P-JNK, P-p53 (Ser15), p21, CyclinD1 and CyclinE by Pu-erh tea extract. Our results indicate that Pu-erh tea water extract inhibits cell proliferation of MDA-MB-231 cells through the induction of apoptosis and the stimulation of cell cycle arrest, which is mediated via activation of the JNK-related pathway.

## Introduction

Accumulating studies of chemo-preventive agents have shown that a diet rich in fruits and vegetables could reduce 7–31% of all cancers worldwide (Sharma et al., 2012). In fact, over 25% of pharmaceutical drugs used today are derived from plants. Currently, four classes of plant-derived anticancer compounds are in clinical use: vinca alkaloids, epipodophyllotoxins, taxanes, and camptothecin derivatives (Shebaby et al., 2014). Tea in China is considered a natural healthy beverage with a 3000-year history. Chen Zang, a famous pharmacist of the Tang Dynasty (618-907 A.C.), highlighted the broad range of health-promoting effects: “Every medicine is the only medicine for a specific disease, but tea is the medicine for all disease” (Wolfram et al., 2006). During the last three centuries, many specialists and scholars have intensely researched the effects and low toxicity of tea (Zheng et al., 2014). The use of tea as a cancer chemopreventive agent has been proposed in the last 20 years (Zhao H. et al., 2011). Based on the degree of fermentation, tea is classified into four major categories: non-fermented green tea, partially fermented oolong or paochong tea, fully fermented black tea and post-fermented Pu-erh tea (Way et al., 2009; Zhao H. et al., 2011). Pu-erh tea [*Camellia assamica* (Mast.) Chang] is produced through a unique microbial fermentation process from the sun-dried leaves of large-leaf tea species in the Yunnan province of China and is widely consumed in southeastern Asia due to its unique flavor and potential health benefits (Way et al., 2009; Yu et al., 2014). This special preparation process makes Pu-erh tea unique in terms of its shelf life, as well as its bioactivities.

A number of *in vitro* and animal studies have demonstrated that Pu-erh tea induced apoptosis and growth arrest in human monoblastic leukemia U937 cells, stomach cancer MKN-45 cells (Hayakawa et al., 2001), the human gastric cancer cell line SGC-7901 (Zhao H. et al., 2011), human Hep-G2 cells (Way et al., 2009), human tongue carcinoma TCA8113 cells (Zhao et al., 2014), HT-29 colon cancer cells (Zhao et al., 2013) and others. An *in vivo* study revealed that Pu-erh tea inhibited tumor metastasis by buccal mucosa cancer U14 cells in BALB/c mice (Zhao et al., 2014).

Triple-negative breast cancer (TNBC) is responsible for a disproportionate number of breast cancer- related deaths and has the worst outcome among all breast cancer subtypes. Although several effective treatment options are available, including surgery, radiation, chemotherapy, and endocrine therapy, the mortality rate of patients with breast cancer remains high (He et al., 2014). Epidemiological studies have shown that drinking tea had a potentially preventive effect on breast cancer (Braakhuis et al., 2016; Xiang et al., 2016). However, the growth inhibition activity of Pu-erh tea water extract on breast cancer cell is still inconclusive.

The present study aimed to evaluate apoptosis and cell cycle progression of the human breast cancer cell line MDA-MB-231 after treatment with Pu-erh tea aqueous extract and to elucidate the possible mechanisms of action.

## Materials and Methods

### Preparation of Pu-erh Tea Water Extract

The preparation of Pu-erh tea water extract was described previously by Zhao H. et al. (2011) and Zhao L.J. et al. (2011). Briefly, 10 g of ripe Pu-erh tea leaves was boiled in water for 30 min three times. The supernatant was collected, concentrated, spray dried to powder form and stored at 4°C. The powder was resolved in 1× PBS at 55°C to generate a 100 mg/mL stock solution of Pu-erh tea water extract. The solution was filtered once with filter paper and once with a 0.2 μm filter syringe (Millipore, Billerica, MA, USA). The sterilized Pu-erh tea solution was aliquoted and stored at -20°C. Three major substances in Pu-erh tea water extracts, polyphenol, tea saccharide, and caffeine, account for 33.13, 9.31, and 4.18%, respectively. Tea pigment levels were substantially increased in the fermented tea. Generally, tea pigments consist of theaflavins, thearubigins, and theabrownins, and the theabrownins level of Pu-erh tea extracts is 7.32% (Zhao L.J. et al., 2011).

### Cell Lines and Antibodies

MDA-MB-231 cells were purchased from the Shanghai Cell Institute Country Cell Bank. The cells were cultured in DMEM/F-12 1:1 (HyClone, Novato, CA, USA) supplemented with 10% fetal bovine serum (HyClone, Novato, CA, USA), 1% of 1000 μg/mL streptomycin, 1000 U/mL penicillin (Solarbio, Beijing, China), and 1% NaHCO_3_ at 37°C in an atmosphere of 5.0% CO_2_.

The antibodies used for the western blot analysis were as follows: anti-p21 (1:1000, Abcam, San Francisco, CA, USA), anti-CyclinD1, anti-CyclinE, anti-PCNA, anti-JNK, anti-P-JNK (1:1000, Santa Cruz, CA, USA), anti-p53, anti-P-p53 (Ser15), anti-PARP-1 and anti-β-actin (1:2000, Cell Signaling Technology, Danvers, MA, USA). The JNK-specific inhibitor SP600125 was purchased from MERCK-CALBIOCHEM (Germany).

### Cell Proliferation Assay

For cell proliferation assays, a cell proliferation ELISA kit for BrdU (colorimetric) (Roche Applied Science, Germany) was used as per the manufacturer’s instructions. In brief, cells were seeded in 96-well plates, and the initial cell number was adjusted to 2 × 10^4^ cells per well. Following Pu-erh tea water extract (0, 100, 300, 500, 700, and 900 μg/mL) treatment, the cells were labeled with BrdU for 4 h. Subsequently, anti-BrdU-POD Fab fragments and substrate were added to the medium. The optical density (OD) was determined at 370 nm using a microplate reader. The results were normalized to the control (untreated cells served as a control).

### Colony Formation Assay

Cells in the logarithmic growth phase were digested into a single-cell suspension with a trypsin-EDTA (Solarbio, Tongzhou District, Beijing, China) solution, and then, 2 mL of the cell suspension was seeded onto 6-well plates (NEST, Wu Xi, Jiang Shu, China) at a density of 200 cells/mL. After adhering, the cells were treated with Pu-erh tea water extracts (0, 100, and 300 μg/mL) for 24, 48, and 72 h and then cultured for 15 days. Untreated cells served as a control (0 μg/mL). Thereafter, the cells were fixed in formaldehyde and stained with 10% Giemsa stain (Solarbio, Tongzhou District, Beijing, China). After multiple washes, the plates were air dried and imaged. Individual clones were counted, and statistical analysis was performed.

### Flow Cytometric Analysis of Cell Apoptosis

MDA-MB-231 cells were plated in 6-well plates (NEST, Wu Xi, Jiang Shu, China) and incubated for 24, 48, and 72 h with Pu-erh tea water extracts (0, 100, and 300 μg/mL), and untreated cells served as a control (0 μg/mL). Briefly, the cells were collected and washed with cold PBS. They were then centrifuged and resuspended in 100 μL of binding buffer containing 5 μL Annexin V/FITC and 10 μL 20 μg/mL PI (Sigma–Aldrich, Germany). The cells were incubated for 15 min at room temperature in the dark. A total of at least 10,000 events were collected and analyzed by flow cytometry (BD, LSRFortessa^TM^, San Diego, CA, USA).

### Detection of the Morphological Characteristics of Apoptosis with Annexin V/FITC and PI Staining

Annexin V binds to phosphatidylserine exposed on the outer cell membrane during the early stages of apoptosis. Thus, double staining with FITC-Annexin V (appearing green) and propidium iodide (appearing red) is often used to detect cells undergoing apoptosis (Liu et al., 2011). The cells were treated with or without Pu-erh tea water extracts (0, 100, and 300 μg/mL) for 24, 48, and 72 h. Untreated cells served as a control (0 μg/mL). The MDA-MB-231 cells were washed with PBS and fixed in 100 μL of binding buffer containing 5 μL Annexin V/FITC and 10 μL 20 μg/mL PI (Sigma-Aldrich, Germany). Then, the cells were incubated for 15 min at room temperature in the dark. Changes in the nuclei of cells after staining with Annexin V/FITC and PI were observed by laser scanning confocal microscopy (FV500, Olympus Corporation).

### Western Blot Analysis

MDA-MB-231 cells were treated with various concentrations of Pu-erh tea water extracts (0, 100, and 300 μg/mL) for 24, 48, and 72 h and then lysed in sample buffer followed by denaturation. Protein concentrations were determined with a BCA Protein Assay Kit (Beyotime, Shanghai, China), and untreated cells served as a control (0 μg/mL). The proteins were separated by SDS-PAGE, transferred to nitrocellulose membranes and blocked with 5% BSA (Solarbio, Tongzhou District, Beijing, China). The processed membranes were then incubated overnight at 4°C with primary antibodies. This was followed by an incubation with goat anti-rabbit/anti-mouse secondary antibody conjugated to horseradish peroxidase (1:5000, R&D Systems, USA). Photographs were obtained, and the ODs of the bands were scanned (Image Scanner III, GE) and quantified with Image-J and GraphPad Prism 5.

### Confocal Fluorescence Microscopy

MDA-MB-231 cells (2 × 10^6^) were seeded onto 25 mm square glass cover slips (NEST, Wu Xi, Jiang Shu, China) and incubated for 24 h with Pu-erh tea water extracts (0, 100, and 300 μg/mL), and untreated cells served as a control (0 μg/mL). They were then fixed in 4% paraformaldehyde in PBS for 20 min at room temperature, and after three washes in PBS, the cells were permeabilized with 0.5% Triton-X-100 (Thermo Fisher Scientific, Waltham, MA, USA) in PBS for 15 min. After three washes in PBS, the cells were fixed in 5% BSA in PBS for 30 min at room temperature. The cells were incubated with an antibody against p21 (1:50, Abcam, San Francisco, CA, USA) at 4°C overnight. After three washes in PBST, the cells were incubated with goat Alexa 488 and rabbit IgG (1:200, Invitrogen, Carlsbad, CA, USA). Nuclei were stained by incubating the cells with 6 μmol/L 4, 6-diamidino-2-phenylindole (DAPI) (Thermo Fisher Scientific, Waltham, MA, USA). The cells were observed by confocal fluorescence microscopy (FV500, Olympus Corporation).

### Cell Cycle Analysis

MDA-MB-231 cells were treated with various concentrations of Pu-erh tea water extracts (0, 100, and 300 μg/mL) for 24 h, and untreated cells served as a control (0 μg/mL). Then, the cells were collected and fixed in cold 70% ethanol and stored at -20°C. The cells were then washed and resuspended in cold PBS and were allowed to incubate at 37°C for 30 min with 10 mg/mL RNase and 1 mg/mL propidium iodide (Sigma–Aldrich, Germany). DNA content analysis was performed by flow cytometry (BD, LSRFortessa^TM^, San Diego, CA, USA). The percentage of cells in different cell cycle phases was determined with Cell Quest acquisition software (BD Biosciences, Pharmingen, San Diego, CA, USA).

### Statistical Analysis

All tests and chemical determinations were performed at least in triplicate, and the data are expressed as the mean ± standard error of the mean (SD). Differences within groups were analyzed with repeated measures two-way ANOVA, and *p* < 0.05 was considered to be statistically significant. All analyses were performed using GraphPad Prism5 (GraphPad Software, Inc., La Jolla, CA, USA).

## Results

### Inhibitory Effect on MDA-MB-231 Cell Proliferation

**FIGURE 1:**
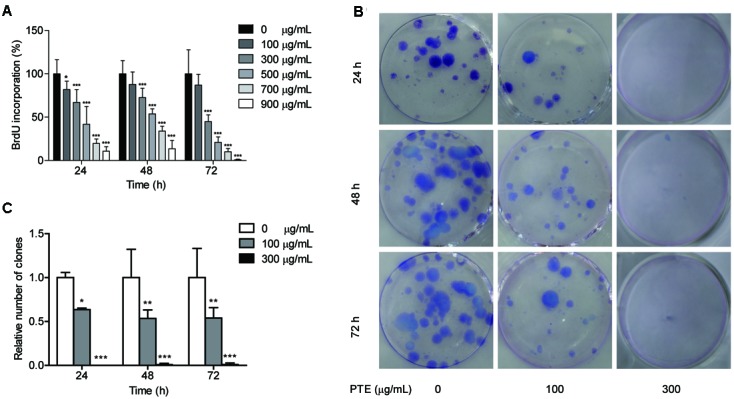
**Pu-erh tea water extract suppressed proliferation of MDA-MB-231 cells. (A)** The effects of Pu-erh tea extract on cell proliferation according to BrdU incorporation assays. MDA-MB-231 cells were incubated with 0–900 μg/mL Pu-erh tea extract for 24, 48, and 72 h. **(B)** Cell viability was detected by colony formation assays. **(C)** The cellular colony formation rates are expressed as a fold change. Results are shown as the mean ± SD. Data are representative of three independent experiments (^∗^*p* < 0.05, ^∗∗^*p* < 0.01, ^∗∗∗^*p* < 0.001).

To determine whether the proliferation of the MDA-MB-231 cells was affected by Pu-erh tea water extract, we measured BrdU incorporation into the cells. Following 24, 48, and 72 h of treatment with Pu-erh tea water extract at various doses, the results of the BrdU incorporation analyses showed that Pu-erh tea water extract dose-dependently decreased the incorporation of BrdU into the MDA-MB-231 cells, as shown in **Figure 1A**. The results showed that after treatment with 100 and 300 μg/mL Pu-erh tea water extract for 24 h, cell viability was decreased by 81.75 and 67.09% (*p* < 0.05), respectively (**Figure 1A**). When the concentration of Pu-erh tea water extract was 454.91 μg/mL, 50% MDA-MB-231 cells were inhibited. These results indicated that Pu-erh tea water extract inhibited MDA-MB-231 cell proliferation.

### Pu-erh Tea Water Extract Suppressed Colony Formation of MDA-MB-231 Cells

The effects of Pu-erh tea water extract on MDA-MB-231 cell growth *in vitro* were tested. As shown in **Figure 1B**, the ability of MDA-MB-231 cells to form colonies in the presence of Pu-erh tea extract was assessed with a flat plate colony formation assay. The colony count indicated that Pu-erh tea water extract resulted in a dose-dependent decrease in the colony formation ability. Moreover, the relative number of colonies of the control were higher than those of the Pu-erh tea water extract-treated group (*p* < 0.05) (**Figure 1C**). The findings support the hypothesis that Pu-erh tea water extract significantly affects the proliferation of MDA-MB-231 cells.

### Effect of Pu-erh Tea Water Extract on Cell Apoptosis and Cell Cycle Arrest of MDA-MB-231 Cells

**FIGURE 2:**
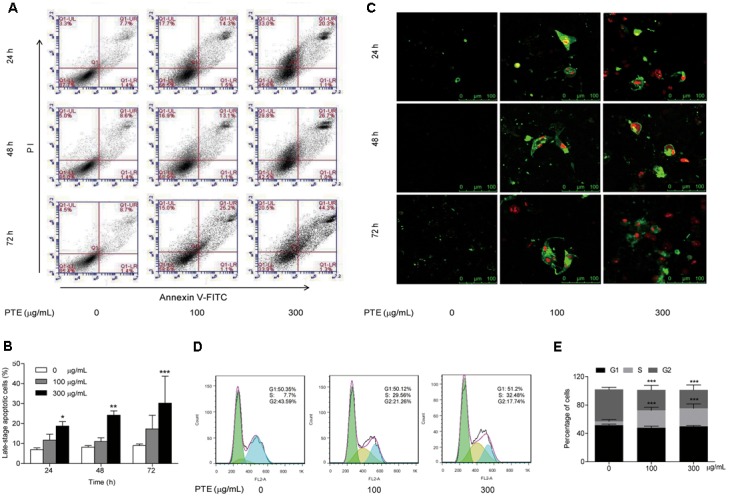
**Effect of Pu-erh tea water extract on apoptosis and cell cycle arrest of MDA-MB-231 cells. (A)** MDA-MB-231 cells were treated with Pu-erh tea extract (0, 100, and 300 μg/mL) for 24, 48, and 72 h and cell apoptosis was determined by flow cytometry. Viable cells are in the lower left (LL) region (negative for both annexin V-FITC and PI). Early apoptotic cells are located in the lower right (LR) region (annexin V-FITC positive). Late apoptotic cells demonstrating extensive cellular and nuclear membrane damage are located in the upper right (UR) region (double positive). Necrotic cells with destroyed cell membrane are in the upper left (UL) region (PI positive). **(B)** The diagram showed the late-stage apoptotic cells percentage of **(A)**. Results are shown as the mean ± SD. Data are representative of three independent experiments (^∗^*p* < 0.05, ^∗∗^*p* < 0.01, ^∗∗∗^*p* < 0.001). **(C)** Cell apoptotic was assessed by Annexin V/FITC and PI staining. Control cells were primarily Annexin V-FITC and PI negative, indicating that they were viable and not undergoing apoptosis. After treatment with Pu-erh tea extract, population of cells were observed to be Annexin V-FITC and PI positive, indicating that they were in late-stage apoptosis (green: stained with Annexin V-FITC, red: stained with PI). **(D)** After treatment with Pu-erh tea water extract (100 and 300 μg/mL) for 24 h, the cells were fixed and stained with propidium iodide, and the DNA content was analyzed by flow cytometry. The results shown are representative of three independent experiments. **(E)** Flow cytometry assays of MDA-MB-231 cells after Pu-erh tea extract treatment for 24 h showed that the cells in S phase were significantly increased, and those in G2 phase were significantly decreased. Results are shown as the mean ± SD. Data are representative of three independent experiments (^∗^*p* < 0.05, ^∗∗^*p* < 0.01, ^∗∗∗^*p* < 0.001).

To determine whether growth inhibition by Pu-erh tea water extract on MDA-MB-231 breast cancer cells was due to apoptosis and cell cycle arrest, flow cytometric analysis and Annexin V/FITC and PI staining were carried out. We observed significant increase in the population of late-stage apoptotic cells in MDA-MB-231 cells when treated with Pu-erh tea water extract (300 μg/mL) (**Figures 2A,B**). The late-stage apoptotic cell populations in MDA-MB-231 cells treated with 300 μg/mL Pu-erh tea water extract for 24, 48 and 72 h, were significantly increased to 18.77 ± 2.24%, 24.23 ± 2.14%, and 31.57 ± 11.66%, respectively, compared to that in the control cells (6.93 ± 0.93%, 8.17 ± 0.84%, and 9.00 ± 0.79%) (**Figure 2B**).

We next evaluated the effect of Pu-erh tea water extract on MDA-MB-231 cell apoptosis using Annexin V/FITC and PI fluorescence staining. We observed Annexin V/FITC (green) and PI (red) signals could barely be detected in control cells, while strong fluorescence densities were visible in response to 100 and 300 μg/mL Pu-erh tea water extract treatment (**Figure 2C**). These results demonstrated that Pu-erh tea water extract induced late-stage apoptosis of MDA-MB-231 cells.

Cell cycle distribution analysis showed that after treatment with Pu-erh tea extract (0, 100, and 300 μg/mL) for 24 h, the percentages of S phase cells were increased from 5.46 ± 2.5% to 24.53 ± 4.39% and 25.5 ± 6.13%, and the percentages of G2 phase cells were decreased from 45.1 ± 2.86% to 28.77 ± 6.62% and 25.83 ± 7.08%. The results showed that the proportion of MDA-MB-231 cells in S phase was significantly increased, while that in G2 phase was significantly decreased, which suggests an S phase arrest induced by Pu-erh tea water extract (**Figures 2D,E**).

### Effect of Pu-erh Tea Water Extract on Proteins Associated with the Cell Cycle in MDA-MB-231 Cells

**FIGURE 3:**
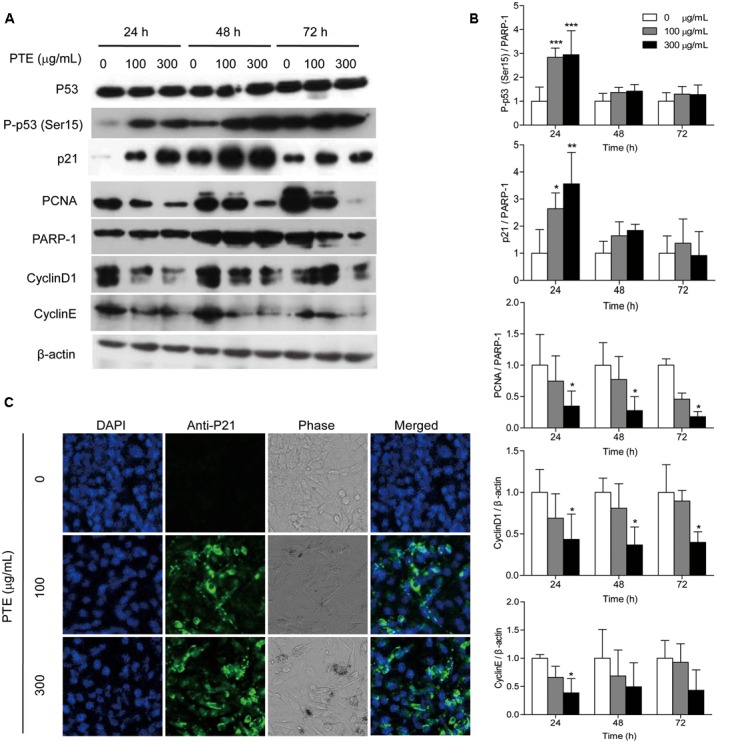
**Pu-erh tea water extract induced the activation of cell cycle-related proteins in MDA-MB-231 cells. (A)** The p53, P-p53 (Ser15), p21, CyclinD1, CyclinE, and PCNA proteins were detected by western blot analysis. PARP-1 and β-actin were used as a loading control. **(B)** Quantification of relative P-p53 (Ser15), p21, CyclinD1, CyclinE, and PCNA protein levels. Columns, average of three independent experiments; bars, SD. ^∗^*p* < 0.05, ^∗∗^*p* < 0.01, ^∗∗∗^*p* < 0.001, significantly different from untreated cells. **(C)** Nuclear localization of p21 protein was determined by immunofluorescence analysis.

To investigate the possible mechanism underlying cell cycle arrest effect of Pu-erh tea water extract on MDA-MB-231 cells, the expression levels of cell cycle-related proteins were assessed by western blot analysis. As shown in **Figure 3A**, treatment with Pu-erh tea extract resulted in the up-regulation of nucleoprotein P-p53 (Ser15) and p21 and the down-regulation of the holoprotein CyclinD1 and CyclinE and the nucleoprotein PCNA at 24 h, which may be partially responsible for the cell cycle arrest of MDA-MB-231 cells. **Figure 3B** shows the relative protein levels of P-p53 (Ser15), p21, CyclinD1, CyclinE, and PCNA. To identify the subcellular localization of p21, which was induced by Pu-erh tea water extract, p21 expression was observed by confocal fluorescence microscopy. p21 was primarily localized to the nuclei of MDA-MB-231 cells treated with Pu-erh tea extract, but it was not detected in the control cells (**Figure 3C**).

### Pu-erh Tea Water Extract Induces Cell Cycle Arrest of MDA-MB-231 Cells via JNK Activation

**FIGURE 4:**
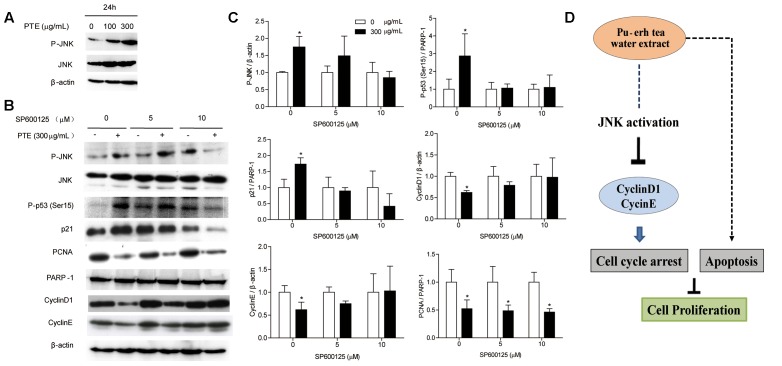
**Pu-erh tea water extract induced cell cycle arrest of MDA-MB-231 cells via JNK activation. (A)** The P-JNK and JNK proteins were detected by western blot analysis. β-actin was used as a loading control. **(B)** The P-JNK, JNK, P-p53 (Ser15), CyclinD1, CyclinE, p21, and PCNA proteins were detected by western blot analysis. β-actin and PARP-1 were used as a loading control. **(C)** Quantification of relative P-p53 (Ser15), p21, PCNA, CyclinD1, CyclinE, and P-JNK protein levels. Columns, average of three independent experiments; bars, SD. ^∗^*p* < 0.05, significantly different from untreated cells. **(D)** Schematic pathway for Pu-erh tea water extract mediating cell cycle arrest and apoptosis in MDA-MB-231 human breast cells.

To determine whether the effect of Pu-erh tea water extract on cell cycle-related protein was due to JNK activation. Therefore, we assessed the expression of JNK and P-JNK in MDA-MB-231 cells after Pu-erh tea water extract treatment by immunoblot analysis. As shown in **Figure 4A**, within 24 h, Pu-erh tea extract increased P-JNK activity in a concentration-dependent manner. We exposed MDA-MB-231 cells to Pu-erh tea water extract and co-treated them with the specific JNK inhibitor SP600125 for 24 h. As shown in **Figures 4B,C**, it was found that after treatment with Pu-erh tea extract, the expression level of P-JNK, P-p53 (Ser15) and p21 were up-regulated and PCNA, CyclinD1, and CyclinE were down-regulated, compared with the control. However, after treatment with Pu-erh tea extract and SP600125, the expression of P-JNK, P-p53 (Ser15), p21, CyclinD1, and CyclinE were restored. It is suggests that JNK activation is important during Pu-erh tea water extract-induced cell cycle arrest. Pu-erh tea water extract inhibited cell proliferation via the induction of apoptosis and cell cycle arrest in MDA-MB-231 cells. This induction of cell cycle arrest occurs through the activation of the JNK-related cell death pathway (**Figure 4D**).

## Discussion

Tea polyphenol is a major cancer-preventive constituent of tea, which has been assessed in many animal models (Yang et al., 2007; Pan et al., 2011). (-)-Epigallocatechin-3-gallate (EGCG), the most abundant polyphenolic compound in green tea, is the prime agent mediating the chemopreventive properities of green tea. *In vivo*, EGCG inhibited tumors in TRAMP mice, a murine transgenic model of prostate adenocarcinoma (PCa) (Modernelli et al., 2015). *In vitro* cell culture studies have shown that EGCG potently induces apoptosis and promotes cell growth arrest through alteration of the expression of cell cycle regulatory proteins, the activation of effector caspases, and the suppression of NF-κB activation (Singh et al., 2011). In this study, we showed that Pu-erh tea water extract inhibited MDA-MB-231 cell growth via stimulation of cell cycle arrest and apoptosis. However, several independent studies have reported that the fermentation process of Pu-erh tea could reduce its total catechins and related anti-oxidant activity, but because Pu-erh tea is produced by promoting enzymatic oxidation of tea polyphenols, we hypothesized that the cell proliferation inhibition components of Pu-erh tea are oxidized tea polyphenols (Zhao L.J. et al., 2011).

As a common beverage in daily life, the toxicity of tea to normal cells should be very limited. A previous report showed that normal cells were not affected by treatment with 500 μg/mL Pu-erh tea water extracts, but this dosage induced early apoptosis in carcinoma cells (Zhao L.J. et al., 2011). In addition, Pu-erh tea water extract (200 μg/mL) could inhibit tumor cell growth by down-regulated S phase and cause G1 and G2 arrest, but at the concentration did not affect wild type mouse embryo fibroblasts (MEFs) growth (Zhao L.J. et al., 2011). In our study, we found that Pu-erh tea water extract significantly inhibited MDA-MB-231 cell growth at 300 μg/mL. At this concentration, Pu-erh tea water extract inhibited tumor cell proliferation by inducing cell apoptosis and cell cycle arrest.

There have been many studies to evaluate the apoptotic status of cancer cells by using Annexin V/FITC and PI fluorescence staining and flow cytometry techniques (Liu et al., 2011; Miao et al., 2013). In our study, we evaluated the effect of Pu-erh tea water extract on MDA-MB-231 cell apoptosis using Annexin V/FITC and PI fluorescence staining, and we observed Annexin V/FITC and PI signals could barely be detected in control cells, while strong fluorescence densities were visible in response to 100 and 300 μg/mL Pu-erh tea water extract treatment. At the same time, by flow cytometry analysis, we observed significant increase in the population of late-stage apoptotic cells in MDA-MB-231 cells when treated with Pu-erh tea water extract. These results demonstrated that Pu-erh tea water extract was capable of inducing MDA-MB-231 cell apoptosis.

We evaluated the effects of Pu-erh tea water extract on cell cycle progression in MDA-MB-231 cells by flow cytometry. We found that the proportion of MDA-MB-231 cells in S phase was significantly increased, while that in G2 phase was significantly decreased, which suggests an S phase arrest induced by Pu-erh tea water extract. A previous study also found that cell cycle progression of the human gastric cancer cell line SGC-7901 was arrested in S phase after treatment with fermented Pu-erh tea (Zhao L.J. et al., 2011). However, many recent studies have demonstrated that MCF7 breast cancer cells treated with EGCG showed G0/G1 phase cell cycle arrest (Khan and Mukhtar, 2008). Pu-erh tea ethyl acetate extract blocked the progression of the cell cycle in HepG2 cells in G1 phase via the induction of p53 expression, which in turn up-regulated p21 expression (Way et al., 2009). Pu-erh tea extract may inhibit osteosarcoma SCID22-3B-1 cell growth by decreasing the proportion of cells in S phase and inducing of G1 or G2 arrest (Zhao L.J. et al., 2011). We hypothesize that the results of Pu-erh tea in tumor cell cycle arrest may be due to the different cell lines and mechanisms.

Accumulating evidence has demonstrated that the role of the JNK pathway in apoptosis is both cell type- and stimulus-dependent (Spurlock et al., 2012; Kim et al., 2014; Liu et al., 2014). Apoptosis may be induced through activation of p53 via JNK and p38 signaling in HT1080 cells (Duan et al., 2011), L929 cells (Sun et al., 2014), and in RA T cells (Spurlock et al., 2012). Here, we also found that in MDA-MB-231 cells, treatment with Pu-erh tea increased the levels of phospho-JNK. Surprisingly, JNK activation was involved in Pu-erh tea water extract-mediated cell cycle arrest, and to determine whether cell cycle arrest is connected to JNK, the JNK-specific inhibitor SP600125 was used. The results showed that SP600125 could block the expression of p21, CyclinD1 and CyclinE. However, PCNA expression did not vary after treatment. p21 is unique among the known CKIs in that it interacts with and inhibits two different targets, the cyclin-CDK complexes that control cell cycle transitions and the DNA polymerase processivity factor PCNA, which is essential for DNA replication. p21 may inhibit cell cycle progression by two independent mechanisms, inhibition of cyclin/CDK complexes and inhibition of PCNA function resulting in both G1 and G2 arrest (Cayrol et al., 1998). Therefore, we hypothesized that the inhibition of CyclinD1 and CyclinE by p21 can result in cell cycle arrest at S phase and inhibition of PCNA resulted in G1 and G2 arrest.

Our data showed that treatment with Pu-erh tea water extract increased the levels of phospho-JNK in MDA-MB-231 cells. Many studies have shown that JNK phosphorylation-induced (2)O(3) c-jun (Ser63/73) can recruit TGIF/HDAC1 to Sp1 binding sites and then suppress p21 promoter activation, blocking cell cycle arrest (Liu and Huang, 2008). Chlorpromazine (CPZ) induction of Egr-1 via the ERK and JNK MAP kinase pathways plays an important role in CPZ-induced p21Waf1/Cip1 expression independent of p53 (Shin et al., 2010). ELK-1 directly *trans*-activates the p21 gene promoter independent of p53. Based on the expression of EGR-1 in NaASO_2_-exposed HaCaT cells (Shin et al., 2011), it is possible that JNK-mediated activation of ELK-1 may contribute to Pu-erh tea-induced p21 expression. In our study, Pu-erh tea water extract inhibited cell proliferation via the induction of cell cycle arrest and apoptosis in MDA-MB-231 cells. This inhibition of cell proliferation occurs through the stimulation of cell cycle arrest, which is mediated via activation of the JNK-related pathway.

## Conclusion

In summary, the present study demonstrated that Pu-erh tea water extract exhibits potent cell proliferation inhibition activity against MDA-MB-231 human breast cancer cells. Pu-erh tea water extract inhibited cell proliferation via the induction of cell cycle arrest and apoptosis in MDA-MB-231 cells. This inhibition of cell proliferation occurs through the stimulation of cell cycle arrest, which is mediated via activation of the JNK-related pathway.

## Author Contributions

JS and H-YZ conceived and designed the experiments. JX, HY, SS, CF, XW, ZB, XM, and SH performed the experiments. JS and H-YZ analyzed the data. H-YZ and JX wrote the paper. All authors reviewed the manuscript.

## Conflict of Interest Statement

The authors declare that the research was conducted in the absence of any commercial or financial relationships that could be construed as a potential conflict of interest.
